# Evaluation of a psychoeducational group program for parents of adolescents with anorexia nervosa: pre-post associations with psychological distress

**DOI:** 10.3389/fpsyt.2026.1847379

**Published:** 2026-09-09

**Authors:** Silvana Giachero, Beate Herpertz-Dahlmann, Brigitte Dahmen, Susanne Gilsbach

**Affiliations:** Department of Child and Adolescent Psychiatry, Psychosomatics and Psychotherapy, University Hospital RWTH Aachen, Aachen, Germany

**Keywords:** anorexia nervosa, eating disorders, parents, psychoeducation, psychological distress

## Abstract

**Introduction:**

Parents play a pivotal role in a child’s or adolescent’s recovery from anorexia nervosa (AN). However, they often experience substantial psychological distress that may limit their ability to support treatment effectively. Structured psychoeducation may help parents understand the illness and treatment process and may support treatment engagement in young people with AN. This uncontrolled pre-post evaluation examined parental psychological distress, self-perceived knowledge and competence, and post-intervention satisfaction with a brief psychoeducational group program.

**Methods:**

Fifty-five parents were screened and 53 consented to participate. Thirty-nine parents of children and adolescents with typical or atypical AN returned complete pre-post data and were included in the analyses. The program consisted of four-session psychoeducational program (90-minute weekly sessions) covering etiology, treatment concepts, nutritional principles, and relapse prevention. Psychological distress was assessed before and after the program using the Brief Symptom Inventory (BSI). Program satisfaction and perceived benefit were evaluated at program completion.

**Results:**

Participants showed high psychological distress at baseline, with significantly lower distress scores after the intervention period. Program satisfaction and attendance were high, with 87% completing all sessions. Parents reported feeling better informed about anorexia nervosa and its treatment, reported high perceived competence in supporting their child and described the material as accessible and understandable.

**Conclusion:**

In this single-center uncontrolled pre-post evaluation, participation in guideline-based psychoeducation was associated with lower parental psychological distress at post-intervention and high acceptability. Because of the uncontrolled design and the absence of patient-level outcomes, these findings should be interpreted as preliminary associations rather than evidence of a causal intervention effect.

## 1 Introduction

Anorexia nervosa (AN) has one of the highest mortality rates among psychiatric disorders (1, 2), and its age of onset has significantly decreased in recent decades (3, 4). AN is characterized by markedly low body weight, severe dietary restriction, preoccupation with body image, and an intense fear of weight gain. In the present study, typical and atypical AN were diagnosed clinically according to ICD-10 criteria used in routine care; atypical AN referred to presentations with one of the diagnostic criteria for AN is not satisfied (not necessarily the weight criterion), for example when body image disturbance is absent (5). This terminology should therefore be distinguished from DSM-5 atypical AN, where patients meet all AN criteria except that weight remains within or above the normal range despite significant weight loss (6).

Since the COVID-19 pandemic, the prevalence of AN and hospital admissions among children and adolescents have risen substantially (7). Several studies also indicate more severe illness trajectories in juvenile AN compared to pre-pandemic samples (8–10). Current treatment guidelines recommend multidisciplinary, coordinated care, including weight restoration, psychoeducation, normalization of eating behavior, and psychotherapy, with strong emphasis on parent and family involvement (11).

Outcomes in adolescent AN are often more favorable than in adults (2), likely due to the active engagement of parents. Adolescents show lower inpatient dropout rates than adults (12, 13), and parents often contribute significantly to post-discharge stabilization.

However, caring for a relative with AN can be highly demanding. In family-based treatment (FBT), for example, parents are explicitly empowered to take an active role in weight restoration and eating support, the eating disorder is externalized from the young person, and responsibility is gradually transferred back to the adolescent as recovery progresses (14). Although such parent-focused approaches may reduce the need for hospitalization or hospital days in some settings (15), they also place a considerable psychological burden on caregivers. Caregiver-burden studies in eating disorders include heterogeneous samples of parents and other caregivers supporting affected relatives both within and outside formal FBT models (16–18). Martín et al. (19) found higher burden levels among caregivers of individuals with AN than among caregivers of those with schizophrenia or depression. Typical symptoms of caregiver burden include depressive and anxiety symptoms (20, 21), guilt, frustration, loneliness (22), and reduced quality of life (23).

Psychoeducation is an established intervention that supports patients and caregivers and has been associated with improvements in caregiver-related outcomes (12, 24) across various disorders (25, 26). In AN, psychoeducation includes information on typical symptoms, the physiological effects of starvation, meal planning, adequate nutrition, relapse prevention, communication strategies, and problem-solving skills (27–29). Its aim is to provide caregivers with knowledge about eating disorders and to support caregivers’ perceived competence and self-efficacy in supporting recovery (27). Geist et al. (26) reported that a psychoeducational intervention for families of individuals with AN achieved weight outcomes comparable to family therapy.

Despite its relevance, few studies have examined psychoeducational programs specifically for parents of children and adolescents with AN (27). In this uncontrolled pre-post evaluation, we examined a psychoeducational group program for parents of patients with typical or atypical AN (ICD-10 F50.0/F50.1) at the Department of Child and Adolescent Psychiatry, Psychosomatics, and Psychotherapy, RWTH University Hospital Aachen.

The primary pre-post outcome was parental psychological distress as measured by the BSI. We additionally examined post-intervention acceptability and satisfaction using the CSQ-8-Adapted and the Final Evaluation Questionnaire, and we described parents’ self-perceived knowledge, competence, and confidence in supporting their child during treatment after participation. Because knowledge and self-efficacy were not assessed with validated pre-post instruments, these constructs are interpreted as self-perceived post-intervention benefit rather than objectively measured change.

## 2 Materials and methods

### 2.1 Psychoeducational group program for parents of patients with AN

Parents involved in the treatment of a child or adolescent with AN or atypical AN were invited and strongly encouraged to participate in the psychoeducational group program as early as possible following the patient’s inpatient or outpatient admission. In this manuscript, the study participants are referred to as parents because all participating caregivers were mothers or fathers; the term caregiver is retained when discussing the broader literature. The psychoeducational group was delivered in parallel with routine multidisciplinary clinical care and was not designed as a stand-alone FBT, emotion-focused family therapy, or enhanced cognitive behavioral therapy (CBT-E) intervention.

In inpatient care, treatment followed a multidisciplinary pathway with the main goals of weight restoration and improvement of eating-disorder psychopathology. This pathway included medical supervision of the patient’s somatic state, nutritional counseling including portion training, psychotherapy based primarily on cognitive-behavioral therapy with FBT elements, treatment of comorbidities, and family involvement through fortnightly parent meetings with the child’s psychotherapist. Crisis meetings were offered when needed, and relapse prevention was addressed with parents during discharge planning. Parent and family sessions were primarily behavior-therapy oriented but incorporated FBT elements, including parents taking responsibility for meal provision with gradual return of responsibility to the patient, and a shift over time from food-related issues to autonomy and adolescence-specific concerns. Outpatient treatment included weekly weight controls, regular blood tests, nutritional counseling, and approximately monthly family sessions with a psychotherapist. Individual meetings with clinicians also took place during the intervention phase. The psychoeducational group itself did not differ between parents of inpatients and outpatients.

The program consisted of four weekly 90-minute sessions and used a standardized slide set. The topics were (1) definition, etiology, and symptoms of AN and atypical AN; physical consequences of starvation and purging behaviors; (2) treatment concept of the department; (3) basic principles of nutritional therapy; (4) relapse prevention. The same psychoeducational intervention was provided to parents of inpatients and outpatients. Sessions were primarily didactic and included time for questions and discussion at the end of each session; participation in discussion was voluntary. Group size averaged approximately 6–7 parents, although group size was not recorded systematically in the study data. Parents did not receive written materials.

**Table 1** summarizes the session-by-session content.

**Table 1:** Session-by-session overview of the psychoeducational group program.

Session	Main topics covered	Key details/goals
1	Diagnosis, epidemiology, etiology, physical consequences of starvation	Diagnostic criteria for AN and AAN; epidemiology; multifactorial etiology (genetic, individual, sociocultural); physical/endocrinological effects of starvation; primary goal: foster understanding of need for weight restoration and relieve relatives’ unjustified guilt
2	Treatment concept and therapeutic techniques	“Four pillars”: 1) Somatic rehabilitation 2) Psychotherapy 3) Family involvement 4) Treatment of comorbidities; target weekly weight gain: inpatient 700 g, outpatient 500 g; psychotherapy addresses body-image and weight-phobia; methods to strengthen ambivalence and cognitive dissonance
3	Nutrition therapy and early stabilization	Role of nutritional therapist; basal metabolic rate (BMR) vs. daily calorie requirements; initial focus on physical stabilization: increase energy intake (start ≥1500 kcal, +200 kcal every 2 days until BMR reached, then adjust to ensure ≥700 g/week weight gain) and limit energy expenditure (restricted activity and school time, rest after meals); practical training (cooking groups, family meals, model-meals)
4	Relapse prevention and aftercare planning	Long-term aftercare importance; gradual return of responsibility for eating/weight to the patient with stabilized behavior; emphasis on age-typical topics (autonomy, friends, hobbies); discharge criteria: maintain target weight corridor for 14 days and sign readmission contract specifying consequences of weight loss (e.g., inpatient readmission); outpatient plan: weekly psychotherapy and weigh-ins, regular labs, half-yearly target-weight adjustments

Sessions on etiology, treatment principles, and relapse prevention were delivered by three clinicians (S.G., S.C.G., and B.D.); the nutritional therapy session was always delivered by the same dietitian. Clinician co-hosted one another’s sessions to support consistency of information. The content was based on the German clinical guidelines for eating disorders (30), evidence-based guidelines of the German Nutrition Society, and international guidelines, including the French Haute Autorité de Santé guideline, the British NICE guideline, the Canadian practice guideline, and the New Zealand guideline on the treatment of eating disorders. [For a comparative overview of international guidelines, see Resmark et al. (31)]. No independent fidelity ratings were conducted.

Session 1 addressed the diagnostic criteria and epidemiology of AN and atypical AN, along with a brief overview of genetic, psychological, and sociocultural contributors to its etiology. Parents received detailed information about the physiological consequences of starvation, including endocrinological and metabolic changes, cardiovascular risk, cognitive and emotional effects of malnutrition, as well as the rationale for weight restoration. The goals were to underline the central importance of weight restoration and to alleviate feelings of shame and guilt often experienced by parents.

Session 2 focused on the department’s treatment concept, structured around four central pillars: (1) physical rehabilitation and medical stabilization; (2) psychological treatment; (3) family involvement, including parents’ supporting role during meals, treatment decisions, and discharge preparation; and (4) management of psychiatric and somatic comorbidities.

In session 3, the nutritional therapist reviewed the aims and practical components of nutritional therapy and weight rehabilitation, including normalization of meal structure, increasing energy intake and supporting eating outside the hospital setting.

In session 4, dedicated to relapse prevention, information on the necessity and structure of long-term aftercare was provided, including weight monitoring, ongoing psychological treatment, nutritional follow-up and communication with outpatient providers. Emphasis was placed on recognizing early warning signs of relapse in childhood and adolescent AN, especially during the vulnerable period following discharge, and on developing coping strategies to support a stabilizing home environment.

### 2.2 Participants

Between May 2022 and November 2023, all parents of children and adolescents with AN or atypical AN who received outpatient, day-care, or inpatient treatment in our department and who participated in the psychoeducational group program were invited to take part in the evaluation study. Inpatient treatment refers to specialized treatment on a child and adolescent psychiatric ward rather than admission to a general medical ward. Psychoeducation for parents of patients with AN is an integral component of our department’s treatment program for all young people diagnosed with AN or atypical AN. Most participants were parents of inpatients (n = 35), while four were parents of outpatients. Inclusion criteria were sufficient German fluency, written informed consent and participation in at least three of four sessions for inclusion in the analyses. No additional exclusion criteria were applied.

The study was approved by the local ethics committee of RWTH University Aachen (reference number EK485-21) and conducted in accordance with the Declaration of Helsinki and Good Clinical Practice guidelines. Written informed consent was obtained from all parents. The study was registered in the German Clinical Trials Register (DRKS00025516).

**Figure 1:**
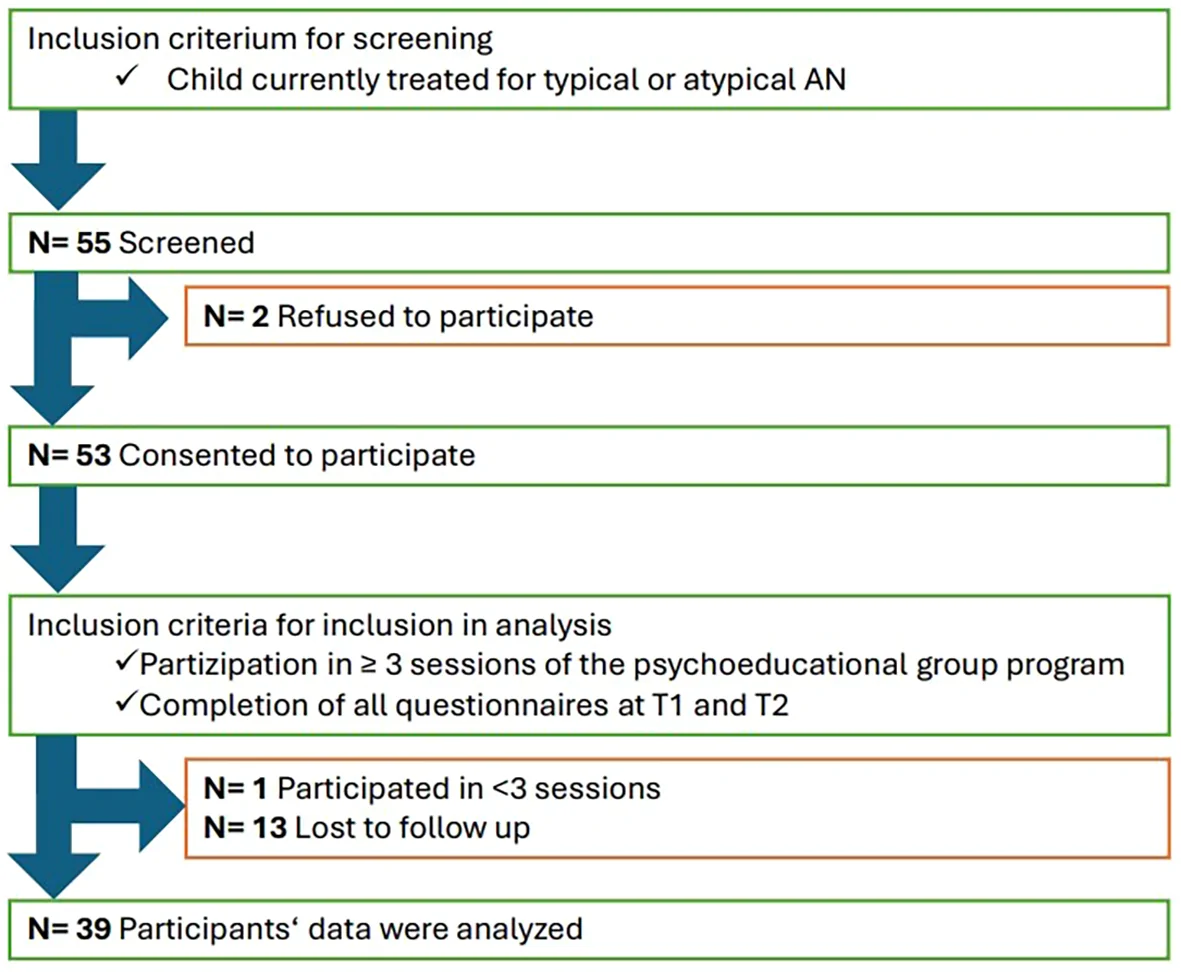
Flowchart of parent recruitment, participation, attrition, and inclusion in the final analyses.

See **Figure 1** for an overview of the recruitment and attrition process.

Among the 39 participating parents, 30 (76.9%) were mothers and 9 (23.1%) were fathers of the patients. The mothers’ average age was 48.5 years (standard deviation (SD): 4.4; range 40–58 years), the fathers’ average age was 51.6 years (SD: 2.7; range 48–57 years). Of the 55 parents screened, 53 consented to participate. Of those, one parent had attended only two sessions of the psychoeducational group program and was therefore excluded; another 13 parents did not return the T2 questionnaires and were classified as lost to follow-up. Thus, 39 parents were included in the final analyses. Of these, 35 were parents of inpatients and four were parents of outpatients. The majority (87.2%) of the analyzed participants completed all four sessions of the psychoeducational group program; 5 parents (12.8%) participated in 3 sessions - the minimum number needed for inclusion in this study. In 5 cases both mother and father of the same patient took part in the psychoeducational group. The study therefore evaluates caregiver-level observations rather than family-level outcomes, and some observations are not fully independent.

The demographic and clinical characteristics of the patients are delineated in **Table 2**.

**Table 2:** Patient characteristics.

N=35 Patients	Number (%) Mean (SD, range)
AN type	
Typical AN	30 (85.8%)
Atypical AN	5 (14.3%)
Sex	
Female	34 (97.1%)
Male	1 (2.9%)
Treatment Setting	
Inpatient	31 (88.6%)
Outpatient	4 (11.4%)
Duration of Illness (months)	12.2 (8.7, 3–39)
Age (y)	14.7 (1.5, 10-17)
Weight at admission (kg)	41.3 (8.6, 27-56)
Height (m)	1.64 (0.07, 1.43–1.78)
BMI (kg/m2)	13.4 (1.9, 11.8–18.7)
BMI-Percentile	3.94 (6, <1-28)

SD, standard deviation; y, years; BMI, body mass index; percentile: age- and sex-adjusted BMI percentile. Sex of parents and patients was reported by parents using a three-option response format (male, female, diverse).

The assessment was conducted before the first session (T1) and after the last session of the group program (T2), after approximately four weeks and only after a minimum of three sessions.

We included only parents in our data analyses who had participated in at least 3 out of 4 sessions of the psychoeducational group program. Missing post-intervention questionnaire data led to exclusion from pre-post analyses; no imputation was performed.

### 2.3 Questionnaires

#### 2.3.1 Brief symptom inventory

The Brief Symptom Inventory (BSI) is a 53-item self-report instrument designed to assess psychological distress experienced by parents during the past seven days across nine symptom dimensions: Somatization, Obsession–Compulsion, Interpersonal Sensitivity, Depression, Anxiety, Hostility, Phobic Anxiety, Paranoid Ideation, and Psychoticism. It also provides three global indices of distress—the Global Severity Index (GSI), the Positive Symptom Distress Index (PSDI), and the Positive Symptom Total (PST)—which quantify overall symptom severity, average symptom intensity, and the number of reported symptoms, respectively (32). Further information, including psychometric properties, is available elsewhere [e.g., (32–34)]. Because item-level BSI responses were not retained in the analysis dataset, internal consistency coefficients could not be calculated retrospectively for the present sample. Previous validation studies have demonstrated good to excellent internal consistency of the BSI. We focused on the three global indices and did not analyze the nine individual symptom dimensions because of the exploratory sample size and the risk of multiple testing.

Items are rated on a 5-point Likert scale ranging from 0 (“not at all”) to 4 (“extremely”). The GSI is calculated by summing all nine symptom dimension scores plus four additional items not assigned to any dimension, and dividing this value by the total number of items completed. The PST represents the number of items with non-zero responses. The PSDI is computed by dividing the sum of all non-zero item scores by the PST and reflects the respondent’s average level of distress.

For the present analyses, raw BSI scores were converted to T scores using the adult normative tables of the German BSI manual. Although the manual provides both total-sample and sex-specific adult norms, the non-sex-specific total-sample norms were used in the present study. Accordingly, **Table 3** reports T scores rather than raw BSI scores. According to the manual’s screening criterion, an individual is classified as showing clinically relevant psychological distress if the GSI T score is ≥ 63 or if at least two of the nine symptom-scale T scores are ≥ 63. Because the present analyses focused exclusively on the three global indices and did not include the nine symptom scales, this screening criterion was used only to contextualize the GSI and was not applied as a clinical cut-off for the PST or PSDI.

**Table 3:** BSI indices at T1 and T2.

N=39 Parents						
BSI index	T1 mean (SD)	T2 mean (SD)	Mean difference (95% CI)	t(df)	p	Cohen’s d
GSI	64.62 (13.57)	55.51 (13.38)	9.10 (5.94–12.27)	5.82 (38)	<.001	0.93
PST	61.10 (12.76)	55.10 (12.91)	6.00 (3.28–8.72)	4.47 (38)	<.001	0.72
PSDI	63.69 (11.21)	54.44 (11.97)	9.26 (5.71–12.80)	5.29 (38)	<.001	0.85

GSI, Global Severity Index; PST, Positive Symptom Total; PSDI, Positive Symptom Distress Index; SD, standard deviation; CI, confidence interval; df, degrees of freedom; t, test statistic; p, p value.

#### 2.3.2 Client satisfaction questionnaire-adapted (CSQ-8-Adapted)

To assess satisfaction with the psychoeducational group program at post-intervention (T2), we administered the CSQ-8-Adapted. This instrument was originally developed to evaluate patient satisfaction with treatment and treatment settings in psychosomatic rehabilitation (35) and was adapted here to assess satisfaction with parent training for parents and other relatives of children and adolescents with eating disorders. The CSQ-8 is a unidimensional measure that provides a homogeneous estimate of overall satisfaction with health care services (35, 36). Each item is rated on a 4-point scale, with higher scores indicating greater satisfaction with the services received. Further information on the instrument, including psychometric properties, can be found elsewhere [e.g., (37)]. In the present sample, the CSQ-8-Adapted demonstrated acceptable internal consistency (Cronbach’s α = .75).

#### 2.3.3 Final evaluation questionnaire

To obtain an overall evaluation of the psychoeducational group program at T2 (end of the program), including self-perceived knowledge and perceived benefit, participants completed a modified version of a questionnaire originally developed by our team after the implementation of psychoeducation several years ago (38). The current version comprises 17 items assessing the perceived benefit of parent training in general as well as for each individual session. Responses are rated on a 4-point Likert scale (from “absolutely not” to “absolutely yes”). Sociodemographic information for parents (age, sex) and clinical characteristics of the patients (weight, height, BMI, age, sex, duration of illness) were collected at the end of the Final Evaluation Questionnaire, separately for fathers and mothers. Sex of parents and patients was reported by parents using a three-option response format (male, female, diverse). The questionnaire was used descriptively and has not been validated as a pre-post measure of knowledge or self-efficacy. Questionnaires were administered at T2 after completion of the program. They were anonymous and completed at home, not in the presence of treatment staff. Because the Final Evaluation Questionnaire was designed as a descriptive program-evaluation instrument covering several distinct domains (e.g., comprehensibility, self-perceived knowledge, and evaluation of individual sessions) rather than a single underlying construct, an overall internal consistency coefficient was not considered informative and was therefore not calculated.

### 2.4 Statistical analyses

For the statistical analyses, we used IBM SPSS Statistics software, version 27.0 for Windows (Released 2020; IBM Corp., Armonk, NY, USA). Data analysis included descriptive statistics (for the Final Evaluation Questionnaire and the CSQ-8-Adapted) and dependent-samples t-tests to compare the three BSI global indices at T1 and T2. Prior to conducting paired-samples t-tests, the assumption of normality of the difference scores was examined using Shapiro-Wilk tests. No significant deviations from normality were detected for the GSI (W = .956, p = .128), PST (W = .971, p = .408), or PSDI (W = .976, p = .575), supporting the use of parametric analyses. Effect sizes were calculated using Cohen’s d for paired samples, and 95% confidence intervals (CIs) were calculated for mean differences. The significance level was set at alpha = .05. To account for testing across the three predefined BSI global indices, a Bonferroni correction was applied, resulting in a corrected significance threshold of p <.017. Missing post-intervention data were handled by complete-case analysis; participants without T2 data were excluded from pre-post analyses and no imputation was performed.

Analyses were conducted at the parent level because the study aimed to evaluate individual caregiver experiences and psychological distress. Although both parents participated in five families, mothers and fathers may differ in their psychological symptoms, and perceptions of the psychoeducational program. Therefore, mothers and fathers from the same family were treated as separate participants. However, responses from caregivers within the same family may be correlated, so observations were not fully independent and the findings should be interpreted with caution. Because the dataset was anonymized after data collection and analyses were conducted at the individual caregiver level, it was not possible to identify paired parents retrospectively. Therefore, a sensitivity analysis excluding one parent per family could not be performed. This limitation should be considered when interpreting the findings.

## 3 Results

### 3.1 Brief symptom inventory

We observed a significant pre-post decrease in parental psychological distress measured by the BSI across all three global indices from T1 to T2. At baseline, the mean GSI T score was 64.62 and therefore exceeded the manual-based threshold of 63, whereas following completion of the psychoeducation program, the mean GSI T score was 55.51 and was below this threshold. Mean PST and PSDI T scores also decreased from T1 to T2. Because this was an uncontrolled pre-post evaluation, these findings indicate an association between participation and lower distress at T2, not a causal intervention effect.

**Table 3** shows the paired t-test for BSI indices (T1 vs T2).

### 3.2 Final evaluation questionnaire

The Final Evaluation Questionnaire (38) indicated high levels of post-intervention perceived benefit among participating parents. Most parents reported feeling better informed about the etiology of AN and strategies for relapse prevention, and they did not experience participation in the psychoeducational group program as burdensome (Question 4). None of the parents considered the information difficult to understand (Question 6). Overall, parents reported that they would recommend the program to others and rated the content as both comprehensible and useful for managing their child’s illness. Percentages were calculated based on valid responses for each item; missing response are reported in the note below **Table 4**.

**Table 4:** Responses to the final evaluation questionnaire (FEQ).

Number (% of valid responses)				
N=39 Parents	Strongly disagree	Disagree	Agree	Strongly agree
The information about the illness was understandable to me.	0	0	18 (46)	21 (54)
The time required for the sessions was appropriate. The division into individual thematic blocks was reasonable.	0	0	21 (54)	18 (46)
The presence of other parents was helpful.	0	6 (16)	24 (65)	7 (19)
Participating in the psychoeducational group program was burdensome.	23 (60)	12 (32)	3 (8)	0
I now understand the treatment better.	0	1 (2)	28 (72)	10 (26)
The information was too technical/difficult to understand.	19 (49)	20 (51)	0	0
The session on “Origins, symptoms and psychological/physical consequences” was particularly interesting to me.	0	3 (8)	17 (45)	18 (47)
I now feel better informed about the origins, symptoms and psychological/physical consequences.	0	2 (5)	22 (56)	15 (39)
The session on “Principles of nutritional therapy” was particularly interesting to me.	0	5 (13)	25 (64)	9 (23)
I now feel better informed about the principles of nutritional therapy.	0	3 (8)	28 (72)	8 (20)
The session on “Treatment concept and modules” was particularly interesting to me.	0	6 (16)	22 (58)	10 (26)
I now feel better informed about the treatment concept and modules.	0	4 (11)	24 (63)	10 (26)
The session on “Relapse prevention and discharge planning” was particularly interesting to me.	0	2 (5)	19 (49)	18 (46)
I now feel better informed about relapse prevention and discharge planning.	0	2 (5)	24 (63)	12 (32)
I had sufficient opportunities to ask questions.	0	0	27 (70)	12 (30)
The group discussion was helpful to me.	1 (2)	5 (14)	25 (70)	5 (14)
The presentation of the topics was didactically well organized.	0	0	29 (76)	9 (24)

Percentages are based on valid responses for each item. Missing responses: “The presence of other parents was helpful” (n = 2); “Participating in the psychoeducational group program was burdensome” (n = 1); “The session on Treatment concept and modules was particularly interesting to me” (n = 1); “I now feel better informed about the treatment concept and modules” (n = 1); “I now feel better informed about relapse prevention and discharge planning” (n = 1); “The group discussion was helpful to me” (n = 3); “The presentation of the topics was didactically well organized” (n = 1).

### 3.3 Overall satisfaction with the psychoeducational group program (CSQ-8)

Post-intervention satisfaction with the psychoeducational group program was also positive, as shown in **Table 5**.

**Table 5:** Responses to the CSQ-8-Adapted.

Number (% of valid responses)				
N = 39 Parents	Largely dissatisfied	Slightly dissatisfied	Satisfied	Very satisfied
How would you rate the quality of the parent education program?	0	0	26 (66.7)	13 (33.3)
Did you get the kind of parent training you were looking for?	0	0	21 (53.9)	18 (46.1)
To what extent did the parent training meet your needs?	0	1 (2.6)	19 (48.7)	19 (48.7)
Would you recommend the parent training to a friend whose child is suffering from anorexia nervosa?	0	0	7 (18)	32 (82)
How satisfied are you with the amount and the kind of advice you received from the parent training program?	1 (2.6)	0	25 (64.1)	13 (33.3)
Did the parent training help you deal with your child’s problems more effectively?	1 (2.6)	0	17 (44.7)	20 (52.6)
How satisfied are you with the parent training program as a whole?	0	0	16 (41)	23 (59)
Would you ask us for advice again if your child needed help?	0	1 (2.6)	5 (12.8)	33 (84.6)

Percentages are based on the number of valid responses for each item. One response was missing for the item “Did the parent training help you deal with your child’s problems more effectively?” (valid n = 38); all other CSQ-8-Adapted items had 39 valid responses.

Half of the items received exclusively positive responses (“satisfied-good” or “very satisfied-excellent”), while the remaining items elicited only a very small number of negative responses (“slightly dissatisfied” or “very dissatisfied”). The four negative responses were provided by three parents, and no association was found between dissatisfaction and any demographic or clinical variables (i.e., parental or child age, patient BMI, or duration of illness). Moreover, no correlation emerged between the CSQ-8 results and BSI scores, suggesting that satisfaction with the program was not closely related to parents’ psychological distress. Although questionnaires were anonymous and completed at home rather than in the presence of treatment staff, satisfaction was assessed shortly after participation; therefore, social desirability and gratitude bias cannot be fully excluded.

## 4 Discussion

Overall, parents reported substantial perceived benefit from the psychoeducational group program. They considered the program helpful and reported feeling more confident in their ability to support their child. Regarding specific sessions, the strongest agreement was expressed for items addressing relapse prevention and preparedness to manage the child’s illness. Moreover, most parents indicated that they would recommend participation in a similar program to other parents. Thus, this inexpensive, time-efficient, and easy-to-implement intervention appears feasible and acceptable in a specialized clinical center. Because patient outcomes were not analyzed, the present data cannot determine whether the program improved weight restoration, eating-disorder psychopathology, treatment adherence, readmission, relapse, or other patient-level outcomes.

One of our aims was to examine whether parental psychological distress changed from before to after the psychoeducational group program and how parents evaluated the intervention. Consistent with previous findings by Holtkamp and Hagenah (38, 39), our sample showed high levels of psychological burden, as measured by BSI scores, at baseline. The BSI measures psychological distress rather than caregiver burden as a multidimensional construct; therefore, the present findings are best described as changes in distress that may be one component of caregiver burden. Notably, the distress in our sample was even higher than that observed in their cohort. Several factors may explain this difference. First, patients in our study were slightly younger (mean age: 14.7 years) than in the sample of Holtkamp and Hagenah (38, 39) (mean age: 15.3 years; range 10.7–19.6 years). Second, our recruitment period (May 2022-November 2023) occurred shortly after the COVID-19 lockdowns, during a time when some restrictions were still in place. Several studies have shown that the COVID-19 pandemic significantly increased parental strain, both in parents of children without eating disorders and in those caring for a child with AN (40, 41). Pandemic-related parental stress could therefore help explain the elevated baseline psychological distress in our sample.

Our findings are broadly consistent with the meta-analysis by Kurnik Mesarič et al. (27), which summarized studies of psychoeducation for children, adolescents and caregivers in the treatment of eating disorders. Across ten studies in which psychoeducation served as either the primary intervention or an active control, the authors reported beneficial outcomes for patients, caregivers, or both. Studies with waiting list controls (24, 29) and the comparison by Geist et al. (26) suggest that psychoeducation may be a useful component of care. However, because our study lacked a control group and was embedded in concurrent clinical treatment, the observed pre-post decrease in distress cannot be attributed solely to psychoeducation.

Our psychoeducational group was designed to enhance parents’ understanding of eating disorders as well as their perceived knowledge, skills, and confidence in supporting their child. In contrast to a similar intervention described by Nicholls and Yi (12), where parents particularly valued the opportunity to share experiences with other caregivers, participants in our program did not consider discussion with other parents to be especially helpful. This difference may reflect contrasts in program structure: our program was more didactic in character, whereas that of Nicholls and Yi blended didactic teaching with parent discussion and reflection.

Importantly, parents in our study expressed strong approval of how the psychoeducational content was delivered. This may have been related to the high attendance observed in the present evaluation. The face-to-face format, delivered in a supportive and nonjudgmental environment, may also have supported attendance and acceptability. By contrast, some studies have reported reduced participation in online psychoeducational programs, including challenges in recruitment (42) and withdrawal following randomization to a web-based intervention (43). Although anonymous home completion of the questionnaires may have reduced pressure to provide positive feedback, immediate satisfaction ratings can still be inflated by social desirability and gratitude bias.

The observed decrease in parental psychological distress is the most clinically relevant association in this evaluation. Several mechanisms may plausibly explain why parents reported lower distress after the program, although these mechanisms were not directly tested. One plausible explanation is that psychoeducation may reduce parents’ uncertainty about anorexia nervosa by improving their understanding of the illness and the rationale underlying evidence-based treatment strategies. A better understanding of the disorder may also alleviate feelings of guilt and self-blame, as parents gain a clearer appreciation of the multifactorial etiology of AN and the reasons for specific therapeutic recommendations. Lower parental psychological distress may in turn help alleviate feelings of guilt in patients with AN, who often perceive themselves as a burden to their families (44). Furthermore, by gaining knowledge about AN and its evidence-based treatment strategies, parents may better understand therapeutic decisions, feel more competent and confident in supporting their child, and provide more effective support during the emotional and behavioral challenges of inpatient or outpatient treatment and weight restoration (39). Improved understanding and increased perceived competence may also reduce behaviors known to maintain eating disorders, such as high expressed emotion or unintentional accommodation of illness behaviors (21, 45). These mechanisms were not directly examined in the present study and should therefore be considered plausible explanations for the observed pre-post association rather than established effects. Future controlled studies including patient-level outcomes are needed to investigate these hypotheses.

Similar findings have been reported beyond the field of eating disorders. Educational interventions for caregivers of children with chronic physical illnesses have been associated with improvements in caregiver mental health, parenting skills, communication, and problem-solving (46). Likewise, parent-focused interventions in autism spectrum disorder and ADHD have been associated with reductions in parenting stress together with higher parental competence and confidence following structured psychoeducation or behavioral parent training (47–51). Although these interventions differ in content and intensity from the present psychoeducational program, they collectively suggest that providing parents with knowledge and practical strategies may support their ability to cope with the challenges of caring for a child with a chronic condition.

Parent-focused interventions in eating disorders differ in intensity and therapeutic targets. FBT aims to empower parents to take an active role in restoring nutrition and may reduce hospitalization in some samples (15). SUCCEAT, ECHO, and FEED/emotion-coaching interventions include psychoeducational components but also add skills training, communication strategies, or emotion-coaching elements; studies have reported feasibility, acceptability, improvements in caregiver outcomes, and in some trials patient-level benefits (52–57). Compared with these more intensive or skills-based models, the Aachen program is shorter, primarily didactic, guideline-oriented, and embedded in routine inpatient or outpatient care. This may make it easier to implement, but it also limits direct comparison with structured family or caregiver-skills interventions.

This study has several limitations. First, the absence of a control group limits causal inference. Although previous studies comparing psychoeducational group programs with non-intervention controls have demonstrated significant benefits (24, 28), it remains possible that parental psychological distress in our sample decreased in part due to concurrent patient treatment rather than because of the psychoeducational intervention alone. This limitation is shared by previous work, including the review by Kurnik Mesarič et al. (27). Second, the evaluation was conducted in one specialized center, which limits generalizability to other healthcare systems, outpatient-only settings, fathers, non-parent caregivers, and culturally different contexts. Third, the final sample may overrepresent motivated or satisfied parents because 13 consenting parents did not return T2 questionnaires and one attended fewer than three sessions. Because demographic and clinical information for parents and patients was collected at T2, parents who did not return T2 questionnaires could not be described demographically and compared with completers. This attrition pattern creates a meaningful risk of selection bias and limits confidence that the analyzed sample is representative of all consenting parents. Fourth, knowledge, competence, and confidence were assessed only as self-perceived post-intervention benefits rather than with validated pre-post measures. Fifth, five families contributed data from both mother and father; these parent-level observations were analyzed independently, which may violate independence assumptions. As noted in the statistical analysis section, a sensitivity analysis excluding one parent per family was not feasible because the anonymized analysis dataset available for this revision did not contain family-level identifiers linked to the BSI outcome data. Sixth, the proportion of all clinically eligible inpatient and outpatient families who participated in the group, the interval from admission or therapy start to the first group session, prior treatment exposure, and amount of pre-admission weight loss were not systematically analyzed in the present dataset. Finally, no patient-level outcomes were analyzed, so the study cannot determine whether the program affected weight restoration, eating-disorder psychopathology, adherence, readmission, relapse, or long-term recovery.

In this uncontrolled pre-post evaluation, the psychoeducational group program for parents of children and adolescents with AN was feasible and acceptable, and participation was associated with lower parental psychological distress at post-intervention. Parents also reported high self-perceived knowledge and competence after participation. Given the uncontrolled design, these results should be interpreted as preliminary pre-post associations rather than causal evidence of program effectiveness. Future controlled research should examine whether similar programs are associated with changes in patient outcomes such as weight restoration, symptom severity, treatment adherence, readmission and relapse. Additionally, evaluating the feasibility and acceptability of delivering the program online or via videoconference would be a valuable avenue for further investigation.

## Data Availability

The raw data supporting the conclusions of this article will be made available by the authors, without undue reservation.
